# Attitudes of transgender men and non-binary people to cervical screening: a cross-sectional mixed-methods study in the UK

**DOI:** 10.3399/BJGP.2020.0905

**Published:** 2021-05-18

**Authors:** Alison M Berner, Dean J Connolly, Imogen Pinnell, Aedan Wolton, Adriana MacNaughton, Chloe Challen, Kate Nambiar, Jacob Bayliss, James Barrett, Christina Richards

**Affiliations:** Centre for Genomics and Computational Biology, Queen Mary University of London; specialty doctor in gender identity medicine, Gender Identity Clinic, Tavistock and Portman NHS Foundation Trust, London.; Geriatric Medicine, Whipps Cross University Hospital, Barts Health NHS Trust; visiting researcher, Institute of Psychiatry, Psychology and Neuroscience, King’s College London, London.; Jo’s Cervical Cancer Trust, London.; Chelsea and Westminster Hospitals NHS Foundation Trust, London.; Jo’s Cervical Cancer Trust, London.; Imperial College London, London.; Gender Identity Clinic, Tavistock and Portman NHS Foundation Trust, London; specialty doctor in sexual health and HIV medicine, Department of Sexual Health, Brighton and Sussex University Hospitals NHS Trust, Brighton.; Brighton and Hove LGBT Switchboard, Brighton.; Gender Identity Clinic, Tavistock and Portman NHS Foundation Trust, London.; Gender Identity Clinic, Tavistock and Portman NHS Foundation Trust, London.

**Keywords:** cervical cancer, gender identity, primary health care, screening, transgender, sexual health

## Abstract

**Background:**

Transgender men and non-binary people assigned female at birth (TMNB) who have not had surgery to remove the cervix are recommended to undertake cervical screening with the same frequency as cisgender women, but evidence suggests that TMNB have lower odds of lifetime and up-to-date cervical screening uptake.

**Aim:**

To understand the attitudes towards and preferences for cervical screening among UK-based TMNB.

**Design and setting:**

Cross-sectional survey of TMNB at an NHS gender identity clinic (GIC) and an NHS sexual health service specialising in care of transgender people.

**Method:**

Recruitment was via email invitations to patients of the GIC and sexual health service. Inclusion criteria were: female sex assigned at birth; transgender man, masculine, or non-binary gender identity; aged ≥18 years; and UK resident. Quantitative results were analysed using descriptive statistics, and free-text comments were analysed thematically.

**Results:**

In total there were 137 participants; 80% identified as transmasculine,18% as non-binary, and the remaining participants reported other noncisgender identities. Sixty-four participants (47%) were eligible for cervical screening and 37 (58%) of those had been screened. Only 34 (53%) of those eligible felt they had sufficient information about cervical screening. Just over half (*n* = 71/134, 53%) stated they would like the option to self-swab for high-risk human papillomavirus. Only half (*n* = 68/134, 51%) of participants were in favour of an automatic invitation for cervical screening. Thematic analysis identified a number of additional barriers to and facilitators of screening.

**Conclusion:**

TMNB have identified numerous potential areas for change that may improve cervical screening uptake and patient experience.

## INTRODUCTION

Cervical cancer is the fifth most common cancer worldwide,^1^ with approximately 98% of cases attributable to malignant transformation of the cervical epithelium in response to chronic infection with ‘high-risk’ human papillomaviruses (hrHPV).^2^^–^^6^ However, it is now largely preventable thanks to cervical screening.^7^ In the UK, this involves the application of a brush to sample ectocervical epithelium, which is tested for hrHPV. If positive, the sample is then examined microscopically for evidence of precancerous changes (cervical cytology).^8^ Cervical screening is recommended for anyone with a cervix every 3 years from age 25–49 years, and every 5 years from age 50–64 years.^9^ Uptake of cervical screening is not uniform across the eligible population, potentially putting some hard-to-reach groups at increased risk of cancer.^10^

Transgender (trans) refers to people who do not identify with the gender assigned to them at birth.^11^ Trans men are birth-assigned females who identify as men or masculine, and non-binary is an umbrella term for the many trans people with gender identities that are neither exclusively male nor female.^11^ Few studies have estimated the size of the trans population.^12^^–^^18^ However, those which have suggest that trans people make up between 0.3% and 1.2% of the worldwide population.^11^^–^^16^ An estimated 200 000–500 000 trans people live in the UK.^17^

Trans men and non-binary people assigned female at birth (TMNB) who have not had surgery to remove the cervix are recommended to undertake cervical screening with the same frequency as cisgender (cis; people who are not trans) women.^19^ There is a small body of US and Canada-based evidence that suggests TMNB have lower odds of lifetime and up-to-date cervical screening uptake, relative to cis women.^20^^–^^23^ Reasons for this disparity are threefold. First, studies exploring cervical screening among TMNB near unanimously suggest that the relationship between trans status and screening need is poorly understood by both patients and their providers, leading to deviation from cis female screening guidelines.^24^^–^^26^ Second, gender-affirming androgen therapy has been associated with increased odds of failing to obtain an adequate cervical cytology sample.^27^ Third, repeated examinations, made technically difficult by androgen-induced changes to vaginal and cervical tissue,^28^ have the potential to bring about significant gender dysphoria and pain for some patients.^29^ Due to a lack of trans status monitoring in healthcare records, estimates of cervical cancer incidence in TMNB are lacking. One US study found a proportional incidence ratio of 0.3 (95% confidence interval [CI] = 0.1 to 0.6) for cervical cancer in trans people compared with cis women, but was unable to stratify trans people according to birth-assigned sex, which is likely to have skewed results.^30^ Further US studies have shown a rate of abnormal cytology of 0.9%–6.7%, and a rate of hrHPV around 16%.^27^^,^^31^^,^^32^

**Table:** How this fits in

The UK’s NHS cervical screening programme has contributed to a significant reduction in cervical cancer mortality since 1988. Transgender men and non-binary people assigned female at birth (TMNB) experience barriers to accessing adequate cervical screening, and are less likely to engage in screening than cisgender women, but the attitudes, experiences, and behaviours of TMNB as they relate to cervical screening remain unexplored in a UK context. This study indicates that TMNB lack sufficient information about cervical screening, and experience barriers to accessing screening services at personal, interpersonal, and institutional levels. Cervical screening uptake could be increased by adopting TMNB-appropriate screening invitations, providing options for self-sampling, improving cultural sensitivity in health literature, and improving access to trans-specific or trans-aware health services.

In the UK, the NHS provides free care at the point of delivery, offers a national call and recall system for cervical screening, and has national clinical guidelines that differ from those in the US. Consequently, the generalisability of findings from the US is uncertain, and, to the authors’ knowledge, there has been no UK-based primary research.^33^ In particular, the UK call and recall system is currently unable to account for those who have registered as male with their GP.^34^ The authors hypothesised that many attitudes of TMNB in the UK would be similar to those demonstrated in international studies, but that there may be unique barriers and facilitators related to the NHS. They undertook a mixed-methods study to better understand the attitudes towards and preferences for cervical screening among UK-based TMNB.

## METHOD

### Study design

This was a cross-sectional questionnaire-based study delivered via the online platform SurveyMonkey (https://www.surveymonkey.com). Participants were asked to disclose demographic information, their attitudes to cervical screening, and their gynaecological, sexual, and cervical screening histories. Optional free-text questions were included for participants to give further detail. The survey was developed in collaboration with Jo’s Cervical Cancer Trust, 56T (a trans-specific sexual health service), and the largest gender identity clinic (GIC) in the UK, based at the Tavistock and Portman NHS Foundation Trust. Participants were drawn from patient populations linked to these organisations.

The final survey comprised 75 questions (Supplementary Table S1); however, the logic of the survey meant that not all questions were administered to all participants.

All responses were recorded anonymously. Participants were made aware that they would be unable to withdraw consent once the survey was submitted because the internet protocol (IP) address storage function of SurveyMonkey had been turned off to ensure anonymity. This anonymisation was to avoid any chance of a participants’ trans identity being inadvertently revealed against their wishes. Participants were reassured that non-response would not affect their care.

Only the findings that relate directly to cervical screening are discussed here. Those pertaining to gynaecological health and healthcare more broadly will be reported subsequently.

### Patient and public involvement

Development of the survey was guided by organisational expertise, literature searching, and patient/public input to focus groups run by Jo’s Cervical Cancer Trust and the LGBT Foundation (a charity working in the UK). The study approach and several drafts of the questionnaire were circulated to the LGBT Foundation, GIC, and 56T for feedback, which was incorporated into the final questionnaire.

### Recruitment

Participants were recruited from the GIC’s waiting list and 56T’s list of TMNB service users at Chelsea and Westminster NHS Foundation Trust sexual health services. Inclusion criteria were: female sex assigned at birth; trans man, masculine, or non-binary gender identity; aged ≥18 years; and UK resident. People held in conditions of security and those lacking capacity were excluded.

A lower age limit of 18 years was selected as it was felt by both the study team and the research ethics committee that it was important to survey the views of those who were approaching, as well as above, the age of screening (25 years in the UK).

Participants were invited by email to participate in the online questionnaire if they had previously given consent to be contacted by email by either the GIC or 56T. Participants were asked to indicate that they had read a study information sheet and fulfilled the eligibility criteria before providing consent electronically and beginning the questionnaire.

The survey was open from 16 August to 7 October 2019. Email invitations were sent to 1304 people. Invitations were staggered to identify any potential issues, with emails sent to GIC patients on 16 August, and invitations to 56T patients sent 2 weeks later. Following survey closure, free-text answers were screened by the chief investigator to ensure any personal identifiable data were deleted before the anonymised results were made available to the full study team. Responders who completed only the screening and consent page, or who did not proceed past demographics questions, were excluded from further analysis. Data quality checks were performed to ensure responses through the survey were logical and consistent. The average time to complete the survey was 12 minutes, and none of the surveys were completed in <4 minutes.

### Data analysis

Statistical analyses were conducted using Microsoft Excel and R (version 3.6.3). Categorical responses were summarised using descriptive statistics, and Fisher’s Exact test was used inferentially to compare responses between groups.

Free-text responses were assessed qualitatively with reflexive thematic analysis, using an essentialist epistemological approach.^35^ Two researchers familiarised themselves with the data, which was stored and analysed using Microsoft Excel. One researcher then coded the data semantically, using both deductive (seeking to answer existing, overarching questions addressed by the questionnaire) and inductive (addressing responses that did not explicitly connect with main research questions but may be relevant to it) approaches. These codes were verified by another researcher. Both investigators acknowledge that their analysis of the data occurs through the lens of a cis clinician–researcher with special interests in sexual health, gender identity, and oncology. Initial themes were deductively constructed by both researchers to address each of the study’s four overarching questions, namely:

- What are the barriers to cervical screening for TMNB?
- What are the facilitators of screening for TMNB?
- How do TMNB want to receive screening information?
- What are the attitudes of TMNB to hrHPV self-sampling?

Further themes emerged inductively from the data, which were felt to map to three additional questions:

- What are the attitudes of TMNB to gynaecological health more broadly?
- What is the range of experiences with gender dysphoria, and how do they relate to cervical screening?
- What are the attitudes to engaging with health care?

Themes were then discussed, agreed, and named. Free-text comments evidencing each theme were recorded. Broader discussion and interpretation of findings of the thematic analysis were carried out within the wider research team, which includes members of the trans community.

## RESULTS

### Sample characteristics

**Figure 1.**
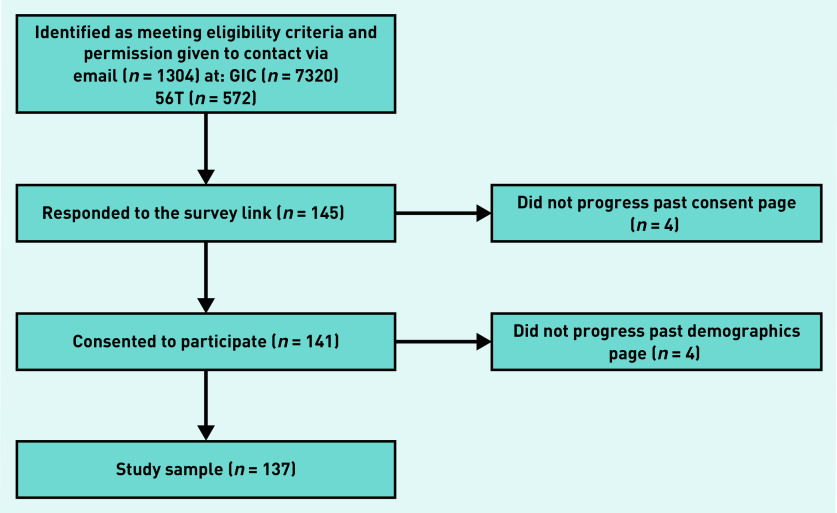
*Schematic of recruitment showing attrition of potential participants from study consent to analysis. GIC = gender identity clinic.*

The survey received 145 responses, with eight participants not progressing past the demographic questions, leaving a sample of 137 (Figure 1). In all, 109 (80%) identified as transmasculine and 24 (18%) as non-binary. The remaining participants reported other non-cis identities, such as ‘non-binary trans man’. The modal age group was 18–24 years. Only 10 (7%) participants were from black, Asian, or minority ethnic backgrounds.

A total of 72% (*n* = 99) were taking masculinising hormones and 7% (*n* = 9/131) had undergone hysterectomy. No participants reported having had cervical cancer. Full demographic information is shown in Table 1.

**Table 1.** Sample characteristics

	***n* ^a^**	**%**
**Age, years**		
18–24	73	53
25–29	35	26
30–34	16	12
35–39	9	7
40–44	1	1
45–49	0	0
50–54	1	1
55–59	1	1
60–64	1	1

**Ethnicity, grouped**		
Asian/Asian British	2	1
Black/African/Caribbean/black British	1	1
Mixed/multiple ethnic groups	6	4
White	127	93
Any other background	1	1

**Country of residence**		
England	132	96
Scotland	0	0
Wales	5	4
Northern Ireland	0	0

**Consider themselves to have a disability**		
Yes	28	20
No	96	70
Prefer not to say	13	9

**Taking hormones**		
Yes	99	72
No	38	28

**Undergone hysterectomy (*n*= 131)**		
Yes	9	7
No	122	93

**Ever attended cervical screening**		
Yes	37	27
No	100	73

a N *= 137, unless otherwise stated.*

### Experiences with, and attitudes towards, cervical screening

Of the 137 participants, 64 (47%) were eligible for screening in the UK (data not shown). Of those eligible, 37 (58%) had ever been for cervical screening (Table 1), and 24 of that 37 (65%) reported having delayed testing at least once. Eight of the 64 participants eligible for screening (13%) had never received an invitation to screen (data not shown).

Only 48 out of 137 (35%) participants felt they had sufficient information about cervical screening and what it might mean for them. Importantly, those eligible for screening felt better informed, with 34 out of 64 (53%) feeling they had adequate information compared with only 14 out of 73 (19%) of those below the age for screening (*P*<0.001) (data not shown).

Though the majority were aware that cervical screening was designed to find precancerous changes in the cervix and that cancer may be rarely detected, only one-third were aware that it was a test to detect HPV, and there were also a variety of misconceptions (Table 2).

**Table 2.** Patient responses to questions on cervical screening

	***n***	**%**
**What do you think cervical screening is? (*n*= 137)**		
A test to find cervical cancer	115	84
A test to find precancerous abnormalities	96	70
A test to check the health of the womb	56	41
A test for human papillomavirus (HPV)	44	32
A test to find ovarian cancer	27	20
A test for sexually transmitted diseases	24	18
A test for chlamydia	10	7
I don’t know	8	6
A test on the health of the cervix	1	1

**If you have not attended cervical screening because of your gender identity, what are the reasons? (*n*= 52)**		
Don’t associate with/like thinking about that part of the body	41	79
How others might react to your gender identity	29	56
Difficult questions	28	54
Disclosure of gender identity	28	54
Triggering dysphoria	2	4
Risk of assault	2	4
Having to be the expert in my own health	1	2
Not wanting the examination by a cisgender healthcare professional	1	2
Being misgendered	1	2
Past bad attitudes to trans people	1	2

**Where would you prefer to attend cervical screening? (*n*= 134)**		
Trans-specific health clinic (any)	86	64
I don’t know	11	8
GP	9	7
Sexual health/GUM clinic	9	7
At home	8	6
I don’t mind	6	4
I would prefer not to attend	2	1
Hospital	2	1
Trans-specific health clinic (discreet)	1	1

*GUM = genitourinary medicine.*

Of 134 participants, 82 (61%) were aware that TMNB registered with their GP as male are not routinely called for cervical screening appointments. This did not differ significantly between those above and below the age for screening eligibility (*P* = 0.86) (data not shown).

**Figure 2.**
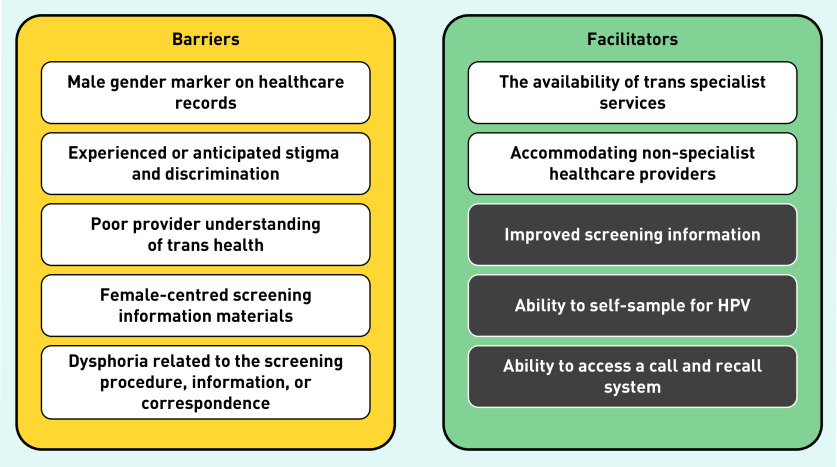
***Barriers to and facilitators of cervical screening in TMNB.*** *^a^* *^a^White boxes indicate primary themes identified and grey boxes indicate additional potential facilitators of screening, given that they assist in overcoming reported barriers. HPV = human papillomavirus. TMNB = transgender men and non-binary people assigned female at birth.*

Having a male gender marker was identified in the thematic analysis as a barrier to screening for TMNB (Figure 2). Consequently, participants were unable to access routine call and recall, or processing of results:

*’*[I] *changed my gender marker to male, so I am not invited at all anymore.’*

(Responder [R] 7020884343)

*’The NHS refused to give me my results as they were under a male gender marker.’*

(R7020898549)

*’I have had trouble booking appointments … receptionists don’t understand.’*

(R7020881677)

Nine participants reported that they had been discouraged from attending cervical screening because of their gender identity, and one was turned away. Almost half (*n* = 30/61) of participants above screening age reported that they chose not to attend cervical screening because of their gender identity (data not shown). Participants’ reasons for not attending screening are shown in Table 2.

These reasons are concordant with further barriers to screening identified in the thematic analysis (Figure 2). For example, ‘difficult questions’ and ‘having to be an expert in my own health’ underline a poor provider understanding of trans health. This is also illustrated particularly well by the experience of one participant:

*’My current GP has never mentioned cervical screening to me in* [the] *years I’ve been at the surgery. I do not feel confident about being trans there.’*

(R7025163064)

Four of the reasons for non-attendance point to experienced or anticipated stigma and discrimination as a major barrier. This was also evident in the thematic analysis:

*’I had met two health professionals … who expressed strong moral objections to the existence of transgender people, and refused to treat me and recommended religion as conversion treatment. I was scared I might encounter someone with a similar mindset who would use a screening as a chance to assault me.’*

(R7025133319)

*’It’s been really difficult to get the nurse/GP to accept me as male, so I think going in and requesting a cervical smear would just take things three steps back and make things more difficult again.’*

(R7020884343)

Finally, dysphoria and discomfort thinking about this part of the body were further reasons related to gender identity that discouraged participants from attending screening. In the thematic analysis, dysphoria could occur:

- in relation to the procedure — *’I can’t stand that part of my body and I don’t want anyone to see it.’* (R7020881854)
- in response to screening information — *’Most of the negative points of the experience came from the very women-focused design and language in the leaflet informing me about it.’* (R7025283699)
- or, as a result of correspondence — *’Documents I have seen … are very feminine and woman/she/her-centred, which would make me uncomfortable if I received them.’* (R7020881966)

### Historical cervical screening behaviour

Twenty-two participants had been for at least one cervical screen since transitioning, and a further 13 had undergone at least one cervical screen before transitioning. The majority of these (*n* = 26/35) were performed at a GP surgery, while four were at a trans-specific health clinic (that is, providing services catering specifically for the needs of trans people). The remaining tests were conducted at a hospital or sexual health clinic. Approximately one-third (*n* = 11/35) of participants reported a negative experience related to their gender identity at their last cervical screening test. One-third (*n* = 11/35) reported a particularly positive experience, and one-third were neutral (*n* = 13/35). The proportion of participants reporting a negative experience was similar between GP surgeries and trans-specific health clinics, though numbers were small (data not shown).

Six of the 35 (17%) participants who were screened reported having an abnormal cervical screening result, and two were invited for colposcopy. Neither reported any negative experience with the colposcopy clinic, and one reported a particularly positive experience (data not shown).

**Figure 3.**
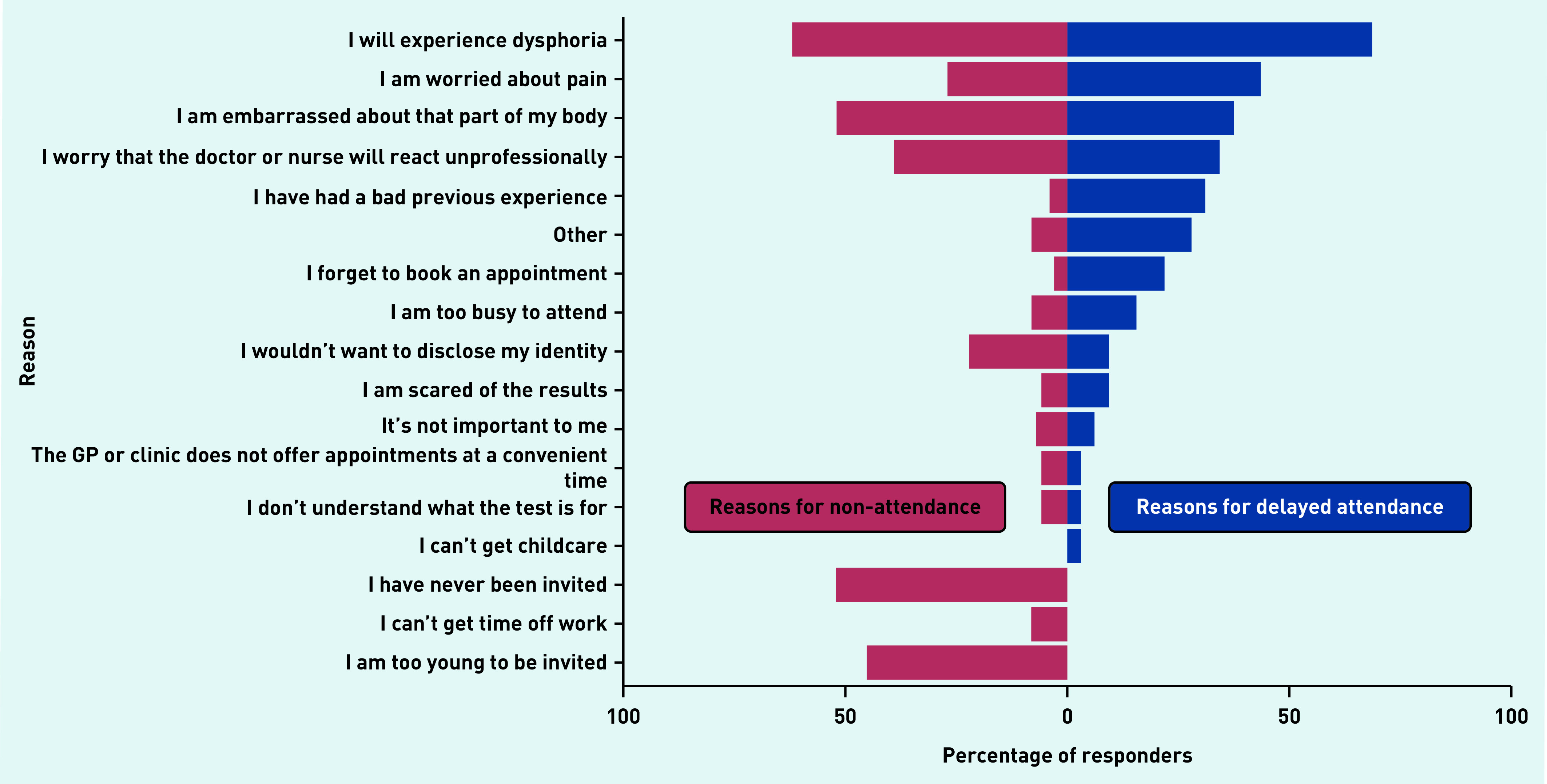
*Reasons for non-attendance for cervical screening in never attenders (*n *= 100) compared with reasons for delaying screening in previous attenders (*n *= 32), by percentage of participants giving each reason.*

Of participants below the eligibility age for cervical screening, 28 out of 73 (40%) stated they would attend once they reached age 25 years. This is lower than for those who had previously attended cervical screening, with 16 out of 35 (46%) saying they would attend again if called, although the result does not reach statistical significance (*P* = 0.14) (data not shown). There were some similarities and differences in the reasons provided for non-attendance in those who had never attended screening and reasons for delaying screening in those who had attended (Figure 3). There were similar levels of concern regarding dysphoria and unprofessional behaviour, echoing the dysphoria and discrimination barriers identified by the thematic analysis (Figure 2). Non-attenders were slightly more likely to report worries about identity disclosure, bodily embarrassment, and problems getting time off work. Attendees who delayed screening were more likely to cite worries about pain, and to state that they forget to book appointments.

### Preferences for future screening

A majority of responders (*n* = 86/134, 64%) stated they would prefer to attend cervical screening at a trans-specific health clinic, with only 7% (*n* = 9) wishing to attend their GP (Table 2). The availability of trans specialist services was a facilitator of cervical screening for TMNB identified by the thematic analysis (Figure 3). These clinics put participants at ease, and had practices that made the screening process easier and less dysphoric:

*’*[They] *sent us home with a speculum so we could practise.* [They] *allowed us to take plenty of time and did brilliant aftercare.’*

(R7020888835)

*’Being a trans clinic, I could ignore my mind and get on with it, knowing they weren’t down there out of curiosity, but just doing their job.’*

(R7025132895)

However, accommodating non-specialist healthcare providers could also be a facilitator of screening:

*’* [The nurse] *could see I was very anxious and worked up afterwards, so she had me sit with her until I was calm and felt comfortable to leave.’*

(R7020888814)

*’My GP was wonderful. I personally have a lot of dysphoria around it. But they were very patient with all of it.’*

(R7020958246)

The gender of the healthcare provider performing the screening was important to 35 out of 134 participants (26%) (data not shown). Some free-text comments discussed a preference for a trans healthcare professional performing their screening.

Self-sampling was a popular alternative to clinician-performed cervical screening. More than half (*n* = 71/134) stated they would like this option, with a further one-third (*n* = 47/134) stating that they needed further information (data not shown).

Thematic analysis of free-text responses regarding self-sampling revealed three key themes. First, participants reported concerns about test efficacy and their personal ability to self-sample. Many participants wanted to receive a self-sampling test only if it was as reliable as the standard clinician-performed cervical screening, while others would choose this option even if it was less reliable:

*’I would need to know … if the procedure is just as good as going to a doctor to have it done. I wouldn’t hold as much trust in doing it at home.’*

(R7020888814)

*’This would be the number one preference; however, it may not be as reliable, which is also acknowledged.’*

(R7020905267)

Some participants felt they would not be able to perform the test because of a lack of expertise or dysphoria, while others felt that, with further information and training, they might be able to do so:

*’I wouldn’t want to muck it up and waste time and NHS money by having to repeat the test by not swabbing correctly.’*

(R7027657737)

Second, the privacy that self-sampling offered participants was a common theme among people who advocated for this option:

*’A partner or spouse could help. I could pick a day and time when I’m feeling less dysphoric, do it in private, know that I’ll be respected and supported, not have to worry about crying in public.’*

(R7021131456)

Third, participants had mixed thoughts about how self-sampling would impact experiences of dysphoria. Some felt that dysphoria would be increased by having to engage with this part of their bodies themselves, whereas others thought it would be reduced by not having to expose this part of their body to another person.

With regards to invitations for screening, 68 out of 134 (51%) would be in favour of an automatic invitation for screening, with a further 34 (25%) unsure (data not shown). In the free-text responses, some participants were in favour of receiving automated or in-person reminders when they were due to screen. The latter could be offered at appointments for androgen administration or when collecting prescriptions. Others, however, stated that reminders would simply serve to increase their dysphoria and would not necessarily increase their likelihood of attendance. Participants expressed concerns regarding both postal and email reminders, as these were deemed possible means to disclose their trans identity. If automated reminders were to be used, many preferred that these come from a trans-specific health clinic that offers appointments for cervical screening. Without automated reminders, only 25 out of 134 (19%) thought that they would remember to attend for screening, with 56 (42%) stating they would not want to go. Only five out of 134 (4%) participants were already arranging their own appointments without reminders (data not shown).

### Factors affecting future cervical cancer risk

Just over half of participants (*n* = 71/131) had received the HPV vaccine, with a further 25 (19%) participants unsure. Of the 35 that had not had the vaccine, 20 (57%) stated they would have it if offered. A further 12 (34%) were unsure. Pregnancy was reported by eight of 131 (6%) participants, and seven of 131 (5%) expressed a wish to become pregnant in the future (data not shown).

### Genital dysphoria and screening

Genital dysphoria was reported by 107 out of 131 (82%) participants. There was broad variation in the ability of participants to have their genitals touched, with around one-third stating this was situation dependent (*n* = 41/131), and a further one-third (*n* = 42/131) stating ‘sometimes’; 24 patients (18%) were able to have their genitals touched, and 17 (13%) were not (data not shown). Thematic analysis confirmed that participants’ ability to have genitals touched in a healthcare setting was variable, with some saying this was more manageable than being touched during sex. Others found it more dysphoric.

### Information materials for cervical screening

Three-quarters of participants (*n* = 101/133) felt that cervical screening materials need to be changed to better reflect the use of services by trans people. Half of participants (*n* = 69/133) expressed a preference for both an information source dedicated to the cervical screening needs of TMNB, and trans-inclusive information in non-specialist screening information materials (data not shown). Most participants wanted reports of real experiences of other attendees, information on emotional support, signposting to LGBTQ+ organisations, and practical tips for attending appointments. Free-text responses identified information to include, such as how to perform the test at home, signposting to trans-friendly providers, and some resources for professionals. Almost all (*n* = 130/133) participants felt that training for healthcare professionals would be useful, and should include general LGBTQ+ awareness training, language/terminology training, and reports from those with lived experience of gender diversity (data not shown).

The thematic analysis explored how participants wanted to receive information on cervical screening. One theme that arose was the use of information sources outwith the NHS and Public Health England (PHE). Social media, specialist trans services, and pride events were identified as more suitable ways to contact and inform the trans community. Another theme related to the divided views on whether cervical screening information materials should be bespoke or shared with cis women. Some quotes illustrating the disparity in responses are shown in Box 1. Common subthemes included the provision of adequate information, lessening of dysphoria, and normalisation of the existence of trans people. Thematic analysis also supported female-centred screening information materials as a barrier to cervical screening.

**Box 1.** Free-text responses exploring whether cervical screening information materials should be bespoke for TMNB or shared with cisgender women^a^

**In favour of bespoke materials**
*’They should not add trans men’s issues to women’s health information. I do not want to be grouped together. I would prefer … a separate version specific to trans men.’* (R7021462140)
*’We exist. To have resources specific to our community … would … help to reduce dysphoria.’* (R7021950777)
*’… more focused on information needed by trans and non-binary people, including statistics … ‘* (R7023896396)
**In favour of shared materials with gender neutral language**
*’I feel uncomfortable accessing information or procedures where I get misgendered.’* (R7025142975)
*’Needs to be more inclusive to normalise trans and non-binary people. It makes trans and non-binary people aware that they should have regular screenings.’* (R7023896396)

a *Quotes have been edited to maintain anonymity. R = responder. TMNB = transgender men and non-binary people assigned female at birth.*

Regarding specific language to use or avoid in screening information, participants were almost unanimous in the need to avoid ‘any “female” terminology’; however, the acceptability of the use of different anatomical terms varied between participants. Importantly, several participants highlighted the need to avoid using possessive terms when referring to genitalia, for example, ‘your cervix’, as this can bring about significant dysphoria.

### Barriers and facilitators to cervical screening

The thematic analysis identified five major barriers and two facilitators to cervical screening in this population, as shown in Figure 2. Though it was not so apparent in the thematic analysis, improved screening information, ability to self-sample for HPV, and the ability to access a call and recall system could also be potential facilitators of screening, given that they assist in overcoming reported barriers and have been identified in other studies.^33^

## DISCUSSION

### Summary

The majority of those eligible had attended for cervical screening, despite many participants reporting that they had insufficient information about the process and what it might mean for them. However, the authors acknowledge that this was a convenience sample, and not necessarily representative of those who do not attend GICs or trans-specific sexual health services. Non-attendance was frequently related to gender identity and, for some, genital dysphoria. This likely contributed to the finding that two-thirds of participants preferred to undergo screening at a trans-specific healthcare clinic.

Half of the sample would like the option to self-sample for hrHPV, with some citing increased privacy and reduced dysphoria as benefits of this approach. However, educational materials would be required to account for the concerns surrounding self-sampling technique and efficacy.

Many participants felt that cervical screening information materials should be adapted to better reflect their use by trans people. Participants wanted dedicated trans-specific resources, as well as a more trans-inclusive approach to non-specialist screening resources. Participants felt that these resources should contain information for professionals caring for trans patients, and nearly all felt that clinicians required further training, particularly surrounding inclusive, appropriate language and general LGBTQ+ awareness.

The gender and experience of the clinician was important to participants, as anticipated, and experienced stigma were reported barriers to attendance. Further barriers included having a male gender marker on electronic health records, and gender dysphoria related to all aspects of the screening process.

### Strengths and limitations

To the authors’ knowledge, this is the first UK-based original research on the perceptions of cervical screening in TMNB, exploring factors unique to the UK, such as the national call and recall system. It involved a comprehensive survey that was broad in scope and included free-text questions to gain a detailed understanding of participants’ experiences. There was stakeholder involvement at each stage, and the team was diverse in experience and identity.

There were several limitations. Despite recruiting from a variety of settings, the response rate was low and the sample small. The sample was limited in geographic and ethnic diversity, and therefore is not representative of the wider trans community. A larger, more representative sample would have been better powered to detect differences between subgroups.

Given that one recruitment centre was a sexual health clinic offering cervical screening and dedicated clinics for trans people, this may have also affected screening rates in the study, as well as contributing to the majority preference for screening at trans-specific sexual health clinics. However, the authors’ staggered invitation strategy means they can confirm that >80% of those who completed the survey were recruited from the GIC, as they completed the survey before invitations were sent for 56T patients.

These recruitment biases mean the authors are unable to compare the rates of screening uptake in this cohort with the published literature,^20^^,^^22^^,^^36^ but they have been able to explore in great granularity the reasons behind screening non-attendance and delay. They strongly advocate for change in screening services and the information materials provided so that participants’ concerns are better addressed.

Though the questionnaire consisted of non-validated measures, it was reviewed by multiple healthcare professionals and third-sector organisations involved in cancer care and LGBTQ+ welfare, including several trans and non-binary people.

### Comparison with existing literature

Several qualitative studies from the US have explored this topic,^24^^,^^29^^,^^37^^–^^39^ but have had smaller sample sizes (most had <50 participants), making this the largest study of this type in trans men and non-binary people, with qualitative data, internationally. These studies have largely corroborated the authors’ findings; however, though these studies have highlighted the role of gender dysphoria in cervical screening behaviour, and suggested it may be more difficult for those with more masculine identities,^29^ the current study suggests greater variability in the relationship between dysphoria and its effects on the individual’s ability to have screening. This highlights the importance of not making assumptions, and of provider awareness. A general desire for trans-specific information sources and evidence-based guidance is also evident in the literature.^24^ Participants who tend to access information online were frequently unclear on which factors influence their cervical cancer risk, including the role of sexual behaviour and androgen use.

High acceptability of self-collected hrHPV swabs among TMNB has been demonstrated in US populations,^37^^,^^38^ with similar concerns to those reported here regarding accuracy and technical difficulties. Other barriers and facilitators to screening identified in the authors’ initial analysis echo those reported in recent literature reviews.^33^^,^^40^ In particular, the availability of trans-specific healthcare services or accommodating, well trained non-specialist providers^33^ can lead to an improved patient–provider relationship, more skilled sample taking, and an improved clinic environment embedded within the wider healthcare system.^40^

A UK-based study with a large sample of cis women highlights that, though some barriers are shared between TMNB and cis women, TMNB experience additional, unique barriers to screening. As with the current study, participants reported bodily embarrassment and difficulty getting time off work for an appointment.^41^ However, unique to the TMNB sample were issues related to gender identity — that is, genital dysphoria and the fear of identity disclosure. Moreover, though preferred screening location was not reported in the study of cis women, the need for a service specialising in minority care was an important facilitator for the authors’ sample.

### Implications for research and practice

General screening information should be gender neutral and applicable to TMNB, with further bespoke information provided that contains accurate, evidence-based recommendations tailored to some of the challenges and misconceptions identified by this study. Such information has now been launched by Jo’s Cervical Cancer Trust.^42^^,^^43^ Healthcare professionals should familiarise themselves with it for their own education, and to direct patients appropriately.

The exclusion of TMNB who are registered male with their GP from the national call and recall system is an obvious barrier to screening. Currently, healthcare professionals taking screening samples must inform the laboratory to return results to their practice rather than the NHS screening service, and take on the responsibility for return of results, colposcopy referral, and time of recall.^34^ The lack of access to standard processes and safeguards could be argued to be systemic discrimination, and places the burden on patients and GPs. The authors urge policymakers to make better provision for TMNB to access the full infrastructure of the screening service, perhaps with the use of a ‘body organ checklist’ (where organs that each patient possesses that are recommended to be screened are recorded), which would also allow patients to opt out of reminders they find triggering. This would need to be coupled with flexible methods of patient contact, and appropriately worded invitations to avoid inducing dysphoria or ‘outing’ individuals. Meanwhile, GPs should ask TMNB if they would like to be included on a reminder register, recognising that not all may wish to do so.^44^ They should also ensure trans awareness training for all practice staff, including receptionists and administrators, and that their information and correspondence is gender neutral. An example of such training is that offered by the LGBT Foundation ‘Pride in Practice’ programme.^45^

This study highlights a demand among TMNB to access cervical screening through specialist clinics, though this may have been affected by how the sample was recruited. The number of NHS clinics providing holistic services that include assessment for gender-affirming treatments, psychology, sexual health, and screening is set to increase,^46^^,^^47^ but they remain limited to major cities, and GPs should be aware of their closest service. Though the authors encourage expansion of such services, they would also welcome initiatives to educate and train GPs and practice nurses in how to approach cervical screening sensitively with TMNB. These should involve partnering with trans-specific sexual health services, as well as patient representatives.

Finally, this study highlights the interest of TMNB in the use of self-collected hrHPV swabs for cervical screening, which may be increased by provision of further patient information. The authors urge PHE to include TMNB in forthcoming screening pilots using hrHPV self-swabs and encourage studies to better determine rates of HPV, abnormal cytology, and cervical cancer in the UK TMNB population, and to tailor healthcare services accordingly.

These findings would be strengthened by a larger study using a nationally representative sample and with adequate advertising to reach TMNB who may not engage with GICs or trans-specific sexual health services. Findings from such a study may also have wider applicability to TMNB in countries with similar healthcare systems.

To appropriately tailor screening services also requires robust estimates of prevalence. For this reason, improved gender identity and trans status monitoring in health care is urgently needed to allow the prevalence of hrHPV and abnormal cervical cytology, and the incidence and mortality of cervical cancer among trans people to be determined. This would also provide the means to generate needs assessments, carry out research, and better design services to meet the healthcare needs of the trans population.
